# Oxygen Mask Related Nasal Integument and Osteocartilagenous Disorders in F-16 Fighter Pilots

**DOI:** 10.1371/journal.pone.0056251

**Published:** 2013-03-07

**Authors:** J. Rieneke C. Schreinemakers, Paul Westers, Pieter van Amerongen, Moshe Kon

**Affiliations:** 1 The Department of Plastic, Reconstructive and Hand Surgery at the University Medical Center Utrecht, Utrecht, The Netherlands; 2 The Department of Biostatistics and Research Support at the Julius Center, University Medical Center Utrecht, Utrecht, The Netherlands; 3 Center for Man in Aviation, Soesterberg, The Netherlands; Harvard Medical School, United States of America

## Abstract

**Background:**

A preliminary survey showed half of the participating Royal Netherlands Air Force (RNLAF) F-16 fighter pilots to have nasal integument and osteocartilagenous disorders related to wearing in-flight oxygen masks.

**Aim:**

To make an inventory of these disorders and possible associated factors.

**Methods:**

All RNLAF F-16 pilots were requested to fill out a semi-structured questionnaire for a cross-sectional survey. Additionally, one squadron in The Netherlands and pilots in operational theater were asked to participate in a prospective study that required filling out a pain score after each flight. Pilot- and flight-related variables on all participants were collected from the RNLAF database. A linear mixed model was built to identify associated factors with the post-flight pain score.

**Results:**

The response rate to the survey was 83%. Ninety of the 108 participants (88%, 6 missing) reported tenderness, irritation, pain, erythema, skin lesions, callous skin, or swelling of nasal bridge integument or architecture. Seventy-two participants (71%, 6 missing) reported their symptoms to be troublesome after a mean of 6±3 out of 10 flights (0;10, 54 missing). Sixty-six pilots participated in scoring post-flight pain. Pain scores were significantly higher if a participant had ≥3 nasal disorders, after longer than average flights, after flying abroad, and after flying with night vision goggles (respectively +2.7 points, p = 0.003; +0.2 points, p = 0.027; +1.8 points, p = 0.001; +1.2 points p = 0.005). Longer than average NVG flights and more than average NVG hours per annum decreased painscores (respectively −0.8 points, p = 0.017; −0.04 points, p = 0.005).

**Conclusions:**

The majority of the RNLAF F-16 fighter pilot community has nasal disorders in the contact area of the oxygen mask, including pain. Six pilot- or flight-related characteristics influence the experienced level of pain.

## Introduction

In 2009, we reported on the external nasal deformities acquired by two F-16 fighter pilots during their flying careers. [1] One pilot's nasal dorsal hump had increased and the overlying skin had become permanently erythematous. The other pilot had developed exostoses on both sides of the nasal bony pyramid. The deformities had been painful after wearing an oxygen mask and sufficiently disturbing to warrant surgery from the pilots' point of view. Subsequent rhinoplasty relieved these symptoms. [1]

We reckoned the in-flight oxygen mask to be the cause of the nasal deformities in both pilots as the oxygen mask may cause tissue trauma by exerting friction and pressure on the nose, each time it is worn, which is during each flight. The nasal problems, however, proved little known to flight surgeons and have, so far, not been reported in medical literature. Therefore, we organized a preliminary inquiry among F-16 fighter pilots in collaboration with the Royal Netherlands Air Force (RNLAF), in September 2008. Half of the thirty pilots who participated reported disorders related to wearing in-flight oxygen masks. To evaluate whether these pilots were a representative sample of the entire RNLAF F-16 pilot community and to determine the spectrum of the nasal disorders, we conducted a cross-sectional survey among all Dutch RNLAF F-16 fighter pilots. To objectify the results of this survey, we additionally conducted a prospective follow-up study that consisted of scoring pain after flying.

## Subjects and Methods

### Ethics statement

We did not seek approval of an Institutional Review Board (IRB) to perform the two studies. At the time the studies were prepared (2008), this survey and the post-flight painscore follow-up study were not regarded as research that required IRB approval, because the research topic was about work-related minor health issues, the questionnaire was short and did not contain loaded or private questions, and scoring post-flight pain took only very little time and did not enforce a change in behavior. Since IRB approval was not required, we also did not obtain IRB approval for the informed consent procedure and we did not obtain verbal or written informed consent. We received permission from the head of operations of the Royal Netherlands Air Force (RNLAF) F-16 fighter pilots to perform both studies. It was granted provided that participation of all participants would be on a voluntary basis, that all participants could stop at any time during the studies, and that the research burden would be limited.

### Cross-sectional survey

#### Subjects and circumstances

All actively flying RNLAF F-16 pilots were considered potential participants for this study. This group included operational squadron pilots and guest pilots. Guest pilots were former operational squadron pilots who had taken on an additional function. All pilots either had the MBU-20/p oxygen mask (GENTEX, Carbondale, PA, U.S.A.), the MBU-12/p oxygen mask (GENTEX, Carbondale, PA, U.S.A.), or both. The majority of the pilots used the MBU-20/p. This oxygen mask consists of a hardshell and a softshell. The softshell is a silicone rubber facepiece. The inside of this facepiece incorporates a reflective edge designed to maintain an airtight seal. [2] The connection between the mask and helmet assembly allows a pilot to put on the mask, pull it more tightly against the face or loosen it. The fitting procedure of the oxygen mask occurs in accordance with the manufacturer's instruction manual. The MBU-20/p oxygen mask is a component of the system that provides pressure breathing for G (PBG) in order to reduce the chance to G-induced loss of consciousness (G-LOCK) [2], but RNLAF F-16 pilots do not make use of PBG. They do receive positive pressure with each inspiration when they reach a cockpit altitude of 28.000 feet or higher from the oxygen regulator. A good fit, therefore, is essential. On the one hand an airtight seal needs to be maintained, on the other hand problems due to a suboptimal fit, such as symptoms of nasal structures and temporomandibular joint symptoms due to retraction of the mandible, need to be prevented. [1]All pilots other than F-16 fighter pilots were excluded to make sure the participants were exposed to the same circumstances as the two F-16 pilots in our case report.

Operations officers requested all F-16 pilots in their squadrons to participate in the cross-sectional survey, but participation was on a voluntary basis. The pilots were informed that the data would be analyzed anonymously.

#### Questionnaire

The cross-sectional survey was executed by use of a semi-structured questionnaire. Because no validated questionnaire for external nasal problems existed, we composed one consisting of a general part and a specific part. The input of the 30 pilots during the preliminary survey was used to fine-tune the questionnaire. The general part asked for demographic data, while the specific part asked for disorders of the nasal osteocartilagenous pyramid or integument. The spectrum of nasal disorders was defined as any change that arises in the oxygen mask area during or following flying. Tenderness, irritation, pain, erythema, skin lesions, callous skin, or swelling of nasal bridge integument or architecture were asked to be reported, including their duration and frequency. We used the arbitrary cut-off point of one week to categorize the symptoms either as temporary or persistent. Additionally, the participants were asked for any steps that had been taken to relieve the disorders (i.e. refitting or adjustment of the mask, in-flight adjustment of the position or removal of the mask, use of blister plasters in the contact area, switching to a different oxygen mask i.e. 12/p or 20/p) and for their willingness to participate in future research to minimize symptoms.

Because we did not want to miss any important, but unexpected information, we encouraged participants to report any information they deemed potentially relevant that was not covered by the questions.

#### RNLAF database information

For each of the participants, pilot-related data on his flying experience (total F-16 flight hours, F-16 flight hours per annum, total flight hours using night vision goggles (NVG), NVG flight hours per annum) were retrieved from the RNLAF database that is continuously updated. We used the arbitrary cut-off point of ≥1.5 hours to categorize flights as long. Flight experience with other aircraft was ignored.

### Prospective study on post-flight pain scores

#### Subjects and circumstances

In addition to the questionnaire, a prospective follow-up study of post-flight pain scores was conducted. To limit the research burden, the RNLAF only allowed the operational squadron and guest pilots at base Leeuwarden in The Netherlands to participate. This part of the study ran for six months, from July 2009 through December 2009. We aimed to include a majority (85%) of the different types of missions flown during this period, which would guarantee exposure to nearly all circumstances that might influence the nasal disorders.

In operational theater in one country abroad, all RNLAF F-16 pilots were allowed to participate irrespective of the bases they were stationed at in The Netherlands. Participation was on a voluntary basis. Only two types of missions were flown in operational theater abroad, 10% of the various types of flights flown in The Netherlands. This part of the study ran from September 2009 through January 2010.

Again, all participants were informed that the data obtained by the pain score would be anonymized.

#### Pain scales

After each flight, the participants indicated whether they had pain in the oxygen mask's contact area with the nose during or after flying using a 0 to 10 numeric rating scale. [3]

#### Additional RNLAF database and personal information

Again, the data on each of the participants' flying experience, and also the characteristics of the flights flown (date of mission, country, duration of mission, duration of NVG use) were retrieved from the RNLAF database, and again flight experience with other aircraft was ignored. Additionally, we used the participants' surveys to retrieve information about severity of nasal disorders, information about possible steps undertaken to relieve them (i.e. including refitting or adjustment of the mask, in-flight adjustment of the position or removal of the mask, use of blister plasters in the contact area, and switching to a different oxygen mask i.e. 12/p or 20/p), and that on the pilot's willingness to participate in future research to minimize the symptoms. The data were assessed as possible associated factors for pain.

#### Data handling

On the basis of the average height of G-forces and the amount of procedural head-movements during standard execution of the flight, two experienced F-16 fighter pilots jointly categorized all the flights flown by the participants in three G-force categories (low, medium, high) and two head movement categories (few, many). Possible disagreement on categorization was resolved by means of discussion until consensus was reached.

### Statistical analysis

All statistical analyses were carried out using spss version 18.0 (SPSS Inc., Chicago, IL, USA). Values of the pilot characteristics are represented as mean (sd) and frequency (%). A multivariable analysis with stepwise model selection was performed in order to assess the optimal combinations of risk factors to explain the post-flight pain score. The whole set of possible risk factors was included. The variables age, total F-16 flight hours, F-16 flight hours per year, duration of a flight in hours, total NVG flight hours, NVG flight hours per year, duration of using NVG, and the number of scored flights in The Netherlands or abroad were assessed as continuous data. The variables operational squadron or guest pilot, using NVG, flights in The Netherlands or abroad in combination with the pilot's base, few or many nasal disorders, low or high G forces during flying, few or many head movements during flying and attempts to reduce disorders were assessed as categorical data. Significance was set at a level of 0.05. To improve the interpretation of the resulting effects of the risk factors and to reduce multicollinearity between the risk factors, the continuous variables (total F-16 flight hours, F-16 flight hours per year, duration of a flight in hours, et cetera) were centered around their mean (i.e. the mean was subtracted from each score). Mean centering does not change the quality of the model. [4] We assumed that the effects of the flight characteristics were the same for every pilot.

We were restricted by the RNLAF in reporting classified military information. Consequently, some of the observations are reported without providing the available, but classified, statistical proof.

## Results

### Cross-sectional survey

#### Participants

One-hundred and eight out of 130 F-16 fighter pilots filled out the questionnaire of the cross-sectional survey, resulting in an over-all response rate of 83% (Table 1). The mean age was 34 (±6), the mean total F-16 flight hours 1456 (±795), the mean total NVG flight hours 84 (±55).

**Table 1:** Characteristics of the 108 participating F-16 pilots in the cross-sectional survey.

Characteristic	Subcategory	mean ± sd (minimum;maximum)	n (%)	Missing
Age		34±6 (24;53)	n.a.	7
Pilot				0
	Operational squadron	n.a.	79 (73%)	
	Guest	n.a.	29 (27%)	
Base				2
	Leeuwarden	n.a.	54 (51%)	
	Volkel	n.a.	52 (49%)	
Total F-16 flight hours		1456±795 (138;3502)	n.a.	3
F-16 flight hours per year		122±62 (0;224)	n.a.	1
Total NVG flight hours^1^		84±55 (0;253)	n.a.	3
NVG flight hours per year		18±14 (0;78)	n.a.	3
Number of nasal disorders				6
	Unknown number of disorders	n.a.	10 (10%)	
	≤2	n.a.	27 (26%)	
	≥3	n.a.	53 (52%)	
Estimated number of flights after which nasal disorders were present		6±3 (0;10)	n.a.	54
Reported flights that brought about or worsened disorders^2^				11
	Long flights (≥1.5 hours)	n.a.	56 (64%)	
	NVG flights	n.a.	61 (70%)	
	Flying abroad	n.a.	17 (20%)	
	Flights with many turns	n.a.	18 (21%)	
	High G forces	n.a.	9 (10%)	
Pilots attempted to reduce disorders				0
	No	n.a.	28 (26%)	
	Yes	n.a.	80 (74%)	
History of nasal trauma		n.a.	3 (5%)	45
ENT nasal surgery		n.a.	8 (13%)	44
No family history of the same nasal disorders		n.a.	99 (100%)	9

1 NVG: night vision goggles. n.a.: not applicable.

2 Several pilots reported more than one of the flight types or combinations such as a long NVG flight abroad.

#### Disorders and their handling

**Figure 1:**
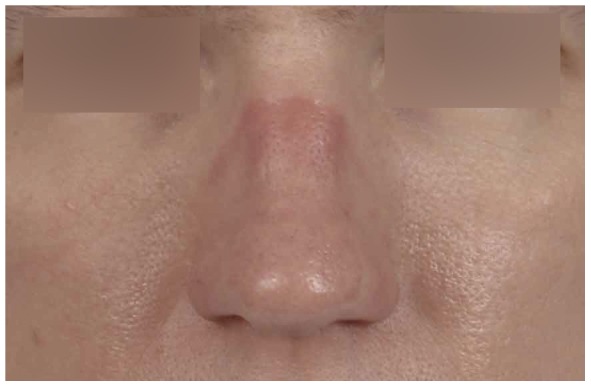
Persistent erythema on the bridge of the nose in the contact area of the oxygen mask.

**Figure 2:**
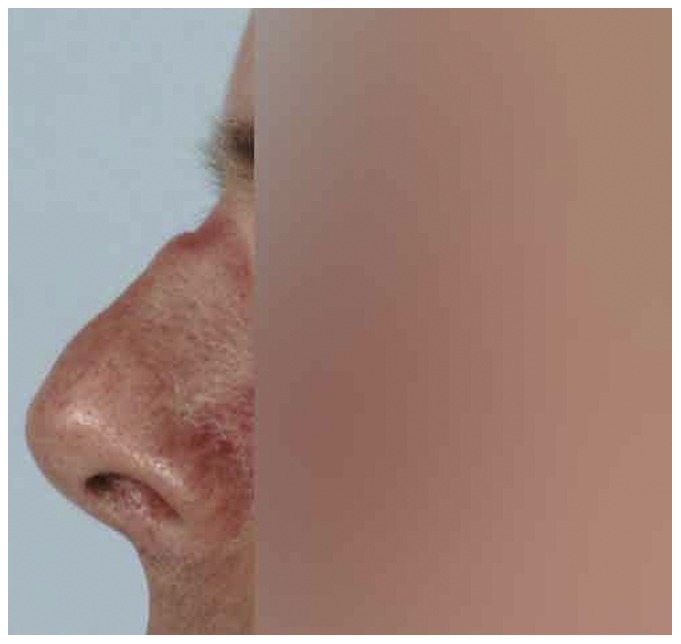
Temporary erythema on the bridge of the nose and an indentation after wearing the oxygen mask.

**Figure 3:**
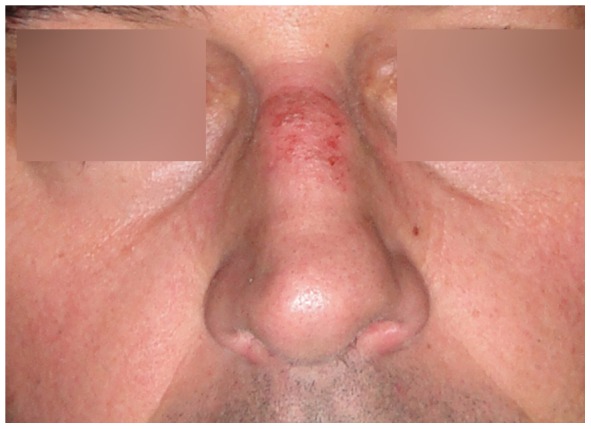
Skin damage after flying with night vision goggles (NVG) for a couple of days in a row.

**Figure 4:**
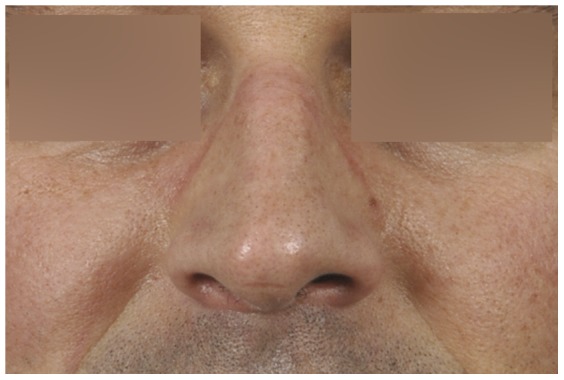
During an NVG-free period the nasal haematoma and swelling are absent in the same pilot (compare with **Figure 3**).

Ninety of the 108 participants (88%, missing data on 6 participants) reported they had, or used to have, tenderness, irritation, pain, erythema, callous skin, or swelling of nasal bridge integument or underlying osteocartilagenous structures (Table 1, Figures 1, 2, 3, 4). Thirty-two of them reported additional symptoms, most of which were skin lesions indicative of exposure to friction and pressure (Figures 1, 2, 3, 4). Their symptoms were located where the oxygen mask presses on the nose, *in casu* the dorsum and sides of the nasal bridge (Figures 1, 2, 3, 4). Seventy-two out of 108 participants (71%, missing data on six participants) reported their symptoms to be troublesome. Fifty-four participants (missing data on 54 participants) reported they had symptoms during or after a mean of six (±3) out of ten flights.

Temporary symptoms were estimated to linger after flying for a median of 12 hours (±20 hours, 0–72 hours) and varied between pilots and between symptoms (missing data on 41 participants). Only 6 participants reported the duration of persistent symptoms, varying from weeks to years.

Eighty-seven participants (missing data on 11 participants) reported which flights gave rise to or worsened symptoms. Fifty-six participants reported long flights (64%), sixty-one reported NVG flights (70%), seventeen reported flying abroad (i.e. long flights, NVG flights; 20%), eighteen flights with many turns (i.e. many head movements; 21%), nine exposure to high G forces (10%). Several pilots reported more than one of the aforementioned flight types or combinations such as a long NVG flight abroad.

Eighty of the ninety participants with disorders (88%) had taken steps to relieve the nasal disorders. Forty-two of them had the flight safety equipment personnel refit or adjust the mask (missing data on 21 participants), which completely relieved only twelve of these forty-two participants of their nasal disorders. Fifty-eight participants adjusted the mask's position during flying, loosened it, or removed the mask when possible (missing data on 25 patients). Twenty-one of the eighty participants had put blister plasters on their noses (missing data on 43 participants). Five participants reported their complaints were resolved by switching from the MBU-12/p oxygen mask to the MBU-20/p or back to the 12/p when the 12/p was being replaced by the 20/p. Eighty-seven of 102 participants (missing data on six participants) were willing to cooperate in research to minimize and prevent possible disorders, whereas one was not. Six additional participants indicated that they might participate, depending on what participation would entail. The eight remaining participants did not have any symptoms.

### Prospective study on post-flight pain scores

#### Participants

Sixty-six F-16 pilots participated in the prospective scoring of post-flight pain. The descriptive characteristics of the participants and the flights were comparable to those of the participants and flights in the cross-sectional survey (Table 2). The mean age was 33 (±6), the mean total F-16 flight hours 1859 (±858), the mean total NVG flight hours 112 (±162). The RNLAF informed us that the response rate for the pilots in The Netherlands was 65% and abroad 95%.

**Table 2:** Characteristics of the 66 participating F-16 pilots and 934 flights in the prospective post-flight pain score study.

Variable	Subcategory	mean ± sd (minimum;maximum)	n (%)	missing
Age		33±6 (24;53)	n.a.	7
Pilot				4
	Operational squadron	n.a.	48 (77%)	
	Guest	n.a.	14 (23%)	
Pilot				0
	The Netherlands, from Leeuwarden^1^	n.a.	42	
	Abroad, from Volkel	n.a.	13	
	Abroad, from Leeuwarden	n.a.	13	
Flights in the Netherlands or abroad, pilot's base				7
	The Netherlands, Leeuwarden	n.a.	530 (57%)	
	Abroad, Volkel	n.a.	228 (25%)	
	Abroad, Leeuwarden	n.a.	169 (18%)	
Number of scored flights per pilot		15±14 (1;52)	n.a.	0
Total F-16 flight hours		1859±858 (398;4170)	n.a.	7
F-16 flight hours per year		138±87 (8;613)	n.a.	7
Duration of a flight in hours		1.7±0.8 (0.2;6.6	n.a.	9
Total NVG flight hours^2^		112±162 (0;1253)	n.a.	7
NVG flight hours per year		21±12 (0;78)	n.a.	7
Duration of using NVG in hours		0.2±0.5 (0;4)	n.a.	1
G forces during flying				0
	Unknown	n.a.	10 (1%)	
	Low	n.a.	721 (77%)	
	Medium	n.a.	119 (13%)	
	High	n.a.	84 (9%)	
Head movements				0
	Unknown	n.a.	9 (1%)	
	Few	n.a.	613 (66%)	
	Many	n.a.	312 (33%)	
Mean of all recorded post-flight painscores		1.8±2.5 (0;10)	n.a.	0
Number of nasal disorders		1.5±2.6 (0;9)	n.a.	9
Pilots attempted to reduce disorders				21
	No	n.a.	5 (11%)	
	Yes	n.a.	39 (89%)	

1 Two pilots participated in The Netherlands and abroad.

2 NVG: night vision goggles. n.a.: not applicable.

#### Pain scores and associated factors

The higher the number of a participant's nasal disorders, the higher the participant's painscores were (+1.8 points if ≤2 disorders, p = 0.068; +2.7 points if ≥3 disorders, p = 0.003, Table 3). For every hour that a flight was longer than the average flight duration of 1.7 hours (Table 2), the pain scores increased with 0.2 points (p = 0.027; Table 3). Participants who did not use night vision goggles had lower pain scores than those who did (−1.2 points, p = 0.005). Participants who used them longer than the average of 0.2 hours (Table 2), and flew more than the average of 21 NVG hours per year (Table 2) had lower pain scores (respectively −0.8 points, p = 0.017; −0.04 points, p = 0.005; Table 3). Participants from Leeuwarden who flew in The Netherlands, had lower pain scores than participants from Leeuwarden who flew abroad (−1.8 points, p = 0.001). The difference between the participants abroad was smaller and not significant (0.5 point, p = 0.290). Participants who had not attempted to reduce the disorders showed a trend of having lower pain scores than those who had (−1.5 points, p = 0.055).

**Table 3:** Effect size, its 95% confidence interval (95%CI) and its p-value of the potential risk factors for post-flight pain as calculated by stepwise multivariable analysis.

Variable	Subcategory	Effect size	p	95% CI of effect size
Flights in The Netherlands or abroad, pilot's base			0.001	
	The Netherlands, Leeuwarden	−1.8±0.3	0.001	(−2.29;−1.28)
	Abroad, Volkel	0.5±0.5	0.290	(−0.45;1.48)
	Abroad, Leeuwarden	0±0	n.a.	n.a.
Duration of a flight		0.2±0.1	0.027	(0.026;0.42)
NVG flight hours per year		−0.04±0.01	0.005	(−0.07;−0.01)
Using NVG^1^			0.005	
	No	−1.2±0.4	0.005	(−2.05;−0.36)
	Yes	0±0	n.a.	n.a.
Duration of using NVG		−0.8±0.3	0.017	(−1.40;−0.14
Nasal disorders			0.007	
	None	0±0	n.a.	n.a.
	≤2	1.8±1.0	0.068	(−0.14;3.68)
	≥3	2.7±0.9	0.003	(0.91;4.39)
No attempt to reduce disorders		−1.5±0.7	0.055	−0.03;2.96

1 NVG: night vision goggles. n.a.: not applicable.

All these factors together explained 56% of the variance in pain between the pilots.

## Discussion

### Current observations

The cross-sectional survey revealed the spectrum of oxygen mask induced nasal disorders in the contact area of the oxygen mask. The vast majority (88%) of the 108 participants of our cross-sectional survey among F-16 fighter pilots had disorders in the nasal contact area of the oxygen mask. The majority of the participants indicated available measures do not relieve these disorders and reported they were willing to participate in future research. They also indicated several factors brought about or worsened the nasal disorders. Subsequently, the post-flight painscore study corroborated that indeed several of these factors including a longer duration of a flight than average, NVG flights and flying abroad induce or worsen pain.

As we expected, the results showed that the pilots with the highest number of nasal disorders, had the highest pain scores. Contrary to what we expected, longer NVG flights than average and a higher than average amount of NVG flight hours per year decreased pain scores. We explain this by regarding more frequent and longer NVG use as chronic exposure to friction and pressure in the nasal area. Pilots may grow accustomed to the pain or experience less pain due to reactive tissue formation, such as the callous skin, the swelling of nasal bridge integument or architecture, and the humps that were reported. [1] We have elaborated on the pathophysiology of the nasal disorders further on in the discussion.

### Methodologic limitations

Before we elaborate on the pathophysiology of these nasal disorders, some potential limitations of our methodology need to be addressed. The cross-sectional design of the questionnaire hampered analyzing how the symptoms develop over time and whether they are correlated to other variables such as a pilot's flying hours. Second, the response rate and the participants' response may have been influenced by the lack of anonymity for the participants. We have tried to minimize response bias by assuring the participants that the information obtained would remain classified and that the data they provided would be anonymized prior to analysis. Third, ours is not a validated questionnaire as it regards a yet non-researched topic. Hence, we are not able to estimate any degree of over- or under-reporting by our participants. Still, we tried to minimize bias by using F-16 pilots' preliminary feedback to rephrase ambiguous questions, and by repeating questions to increase reproducibility.

The prospective study after post-flight pain scores may have been biased as the scoring of pain after flying was part of debriefing and, hence, not always done in private. Another limitation is the unequal distribution of participants over the categories of the categorical variables. We do not know how equal distribution over the categories may change the results. Also, we cannot conclude anything about the effect of using NVG for pilots who did score after NVG flights, but not after flights without NVG and vice versa.

### Pathophysiology of the nasal disorders

In our case-report we reported that the oxygen mask worn by F-16 fighter pilots from the RNLAF may inflict injury to external nasal soft and hard tissues. [1] All of the resulting disorders have separately been reported previously, as such, the acute temporary soft tissue injury observed in F-16 fighter pilots is similar to the discomfort, pain, and skin lesions that have reportedly been caused by oxygen masks used for non-invasive pressure ventilation. [5], [6] Likewise, the persistent erythema reported by our participants may be compared to the telangiectasies that can develop after repeated rhinoplasties. [7], [8] The persistent swellings in the dorsum and sides of the nose presumably caused by chronic exposure of osteocartilagenous stuctures to oxygen mask exerted friction and pressure may be compared to the persistent hard tissue injury observed among swimmers. Swimmers' goggles inflict repetitive trauma during the water re-entry phase of breathing that may lead to development of an asymmetric dorsolateral hump on the nose. [9] The resulting pathophysiology of excessive fibrocartilage formation is similar to that of a cauliflower ear arising from a subperichondrial hematoma after stretching of, or direct trauma to, cartilagenous structures. [10] Finally, the pathophysiology of new bone formation induced by pressure has previously been illustrated in animal studies. Cyclic fatigue loading was shown to cause microdamage of bones with subsequent new bone formation. [11], [12]

We feel that the disorders may easily arise in the nasal bridge area because the integument overlying the nasal osteocartilagenous pyramid is thinner than that elsewhere in the face. [13] Consequently, the exerted friction and pressure have a relatively higher impact. The oxygen mask may cause soft tissue trauma by the friction between the mask and the nose that is caused by movement of the mask. Each time the mask moves over the skin due to G, a suboptimal fit, or movement of the helmet mask assembly, friction may arise. The oxygen mask may also cause nasal trauma by the pressure it exerts. Even though we do not know when disorders tend to arise, it is clear that the nose is chronically exposed to friction and pressure, namely each time the oxygen mask is worn Since the pilots start wearing the same types of in-flight oxygen masks that are worn in an F-16 already in military flying school, and it takes about 375 hours of flight training, including 100 hours of flying an F-16, before one becomes an operational F-16 fighter pilot, it takes approximately 5 to 8 years after training to collect the participants' mean number of 1456 flying hours. Therefore, the participants were chronically exposed and may have developed nasal symptoms, in some worsening up to the point of developing permanent disorders or deformities. In further research, we hope to elaborate on the trauma inflicted by the oxygen mask and the associated specific nasal disorders.

### Implications of our observations

To date, little attention has been given to oxygen mask-induced external nasal symptoms in F-16 fighter pilots. One explanation may be that fighter pilots do not complain easily. Another reason may be that the majority of pilots grow accustomed to the irritation and pain, perhaps as a result of the integumental and osteocartilagenous changes in response to the friction and pressure. A third explanation may be that the types of missions that are frequently flown have changed over the years. The mounting of NVG on the front of the helmet, for example. This shifts the point of gravity forwards and leads to forward rotation of the helmet. [14], [15] Because the mask is held in position by its bayonet-receiver connection to the helmet, this forward rotation of the helmet also changes the position of the mask on the face and probably increases the exerted pressure in the nasal area. Unfortunately, counter balance weights as used in helicopter pilots to reduce helmet slippage cannot be used in fighter pilots, because the latter are exposed to high gravity acceleration. NVG flights are more frequently flown than a couple of years ago. Symptoms, therefore, may have worsened and become more widespread. Furthermore, the fact that our model explains 56% of the variance between the participants makes it highly likely that we have not uncovered all contributing, let alone causative, factors of the nasal disorders. Further research, therefore, is warranted.

## Conclusions and Recommendations

We conclude that the majority of the RNLAF F-16 pilots have nasal integument and osteocartilagenous injuries induced by in-flight oxygen masks. Several pilot- and flight-related characteristics are associated with post-flight pain. The oxygen mask induced nasal injuries are a relevant work-related health issue for RNLAF F-16 fighter pilots.

We recommend that further research is initiated to learn how to prevent oxygen mask induced nasal problems.
